# Early-Branched Short Renal Arteries Are False Multiple Renal Arteries

**DOI:** 10.3390/diagnostics15081046

**Published:** 2025-04-20

**Authors:** Adelina Maria Jianu, Nawwaf Sebastian Damen, Monica Adriana Vaida, Laura Octavia Grigoriță, Marius Ioan Rusu, Mugurel Constantin Rusu

**Affiliations:** 1 Department of Anatomy and Embryology, Faculty of Medicine, “Victor Babeș” University of Medicine and Pharmacy, 300041 Timișoara, Romania; adelina.jianu@umft.ro (A.M.J.); vaida.monica@umft.ro (M.A.V.); grigorita.laura@umft.ro (L.O.G.); 2 Division of Economic Informatics, Faculty of Cybernetics, Statistics and Economic Informatics, University of Economic Studies, 010374 Bucharest, Romania; rusumarius21@stud.ase.ro; 3 Division of Anatomy, Department 1, Faculty of Dentistry, “Carol Davila” University of Medicine and Pharmacy, 020021 Bucharest, Romania; mugurel.rusu@umfcd.ro

**Keywords:** kidneys, anatomical variation, aorta, renal hilum

## Abstract

**Background/Objectives**: During retroperitoneal surgery, awareness of the anatomic variants of renal arteries (RAs) is essential. We aimed to determine the prevalence of early-branched (short) Ras, the bilateral morphologies of RAs in such cases, and to check for significant correlations regarding gender or side. Short RAs may be regarded as false multiple RAs and should be distinguished from true RAs. **Methods**: For the study, 185 archived angioCT files were randomly selected and evaluated for <1.5 cm RAs (106 male and 79 female cases). Simple regression and multiple regression tests, alongside ANOVA, were used for the statistical analysis. **Results**: Short RAs were found in 15/185 cases, 12 males and 3 females (8.1%), with short RAs found on the right side (2.7%), left side (4.86), and bilaterally (one case, 0.54%). The mean length was 9.46 mm. Short RAs were bifurcated in most cases and trifurcated in one case. In four other cases, peculiar RA anatomical patterns were found. They included a right RA origin of the right inferior phrenic artery, variable polar RAs, malrotated and ptotic kidneys, anteriorly dehiscent renal sinuses, and multiple RAs, including five right RAs, with the three inferior ones having precaval courses. Short RAs were not significantly related to gender (*p* > 0.05). There was a significant correlation between gender and right short RAs (*p* < 0.05). **Conclusions**: During renal transplant surgery, distinguishing between true and false multiple RAs is essential. While true multiple RAs may cause surgical discomfort, short RAs may be used as single RAs, but they should be carefully documented before donor nephrectomies.

## 1. Introduction

The renal vascular pedicle typically consists of a renal artery (RA) with aortic origin and a renal vein (RV) ending in the inferior vena cava [1,2]. However, the variations in renal vessels are diverse [3,4,5,6].

The division point of an RA into two or more consecutive branches of at least 3 mm in diameter defines the end of a main RA [7]. Few previous studies have dealt with the length of the main RAs [7,8]. In 1000 cases, the main right RA (RRA) was found to be longer than the left one (LRA) (+6 mm, *p* < 0.001) [7]. In that study, the length of the LRA was 34.8 ± 12.5 mm, and the length of the RRA was 41.4 ± 15.0 mm [7].

Bergman’s Encyclopedia of Human Anatomic Variation [9] quotes just two studies in which the length of RAs is indicated: (1) a dissection study by Ross et al. (1961) on just 34 bodies, in which the average length of the RRA was 39 mm (min. 8 mm, max. 81 mm) and that of the LRA was 33 mm (min. 9 mm, max. 58 mm) [8]; and (2) a study by Hazirolan et al. (2011), which is a pictorial review, where the authors did not measure the renal vessels and just wrote that RAs “are usually 4–6 cm in length and 5–6 mm in diameter” [10]. Satyapal et al. (2001) measured the lengths of accessory RAs [11]. In that study, the lengths of the main RAs were not evaluated.

The branches of an RA may have prehilar (81.67%), hilar (10%), or intrasinusal origins (8.33%) [12]. Early branching of RAs is significant because a long undivided artery is preferable for a kidney transplant [13]. Recto et al. (2019) documented that early branching of RAs was described in as few as 21/64 articles, but no universal definition was used; some of them used an arbitrary limit of 1.5 or 2 cm between the aorta and the division of the main RA in multiple branches, while some others did not specify a limit [14]. These authors did not document whether or not significant correlations regarding early-branched RAs were found in those studies [14]. Bilateral patterning of early-branched (short) RAs was seemingly not done.

We therefore decided to determine the prevalence of such early-branched (short) RAs and the bilateral morphologies of the RAs in those cases and attempt to establish whether significant correlations regarding gender or side could be found. Short RAs may also be regarded as false multiple RAs. During retroperitoneal surgery, it is crucial to distinguish false RAs from true RAs.

## 2. Materials and Methods

For the study, 185 archived angioCT files were randomly selected. There were 106 male and 79 female cases, with a mean age of 68. The inclusion criteria were good-quality scans, complete filling of the abdominopelvic vessels with contrast substance, and no pathological processes distorting the renal vascular anatomy. The exclusion criteria were previous retroperitoneal surgery, pathological processes that distorted the vascular anatomy, and incomplete or unclear angioCT scans. No cases were excluded. The research followed the principles of the World Medical Association Code of Ethics (Declaration of Helsinki). The Ethical Committee of the “Victor Babeş” University of Medicine and Pharmacy of Timişoara, Romania (affiliation 1) approved the study (approval no. 16178/11 July 2023).

A 32-slice scanner (Siemens Multislice Perspective Scanner, Forcheim, Germany) was used for the CT angiograms with previously described technical parameters [15]. The cases were documented using the Horos 3.3.6 (Horos Project, Annapolis, MD, USA) program, as in other studies [5]. All of the authors independently performed evaluations of the renal vascular pedicles. The positive results were identical and were validated by each author. The cases with RAs <1.5 cm in length were recorded and documented individually.

Simple regression and multiple regression tests, alongside ANOVA, were used for the statistical analysis to study the correlations and relationships between various parameters. Microsoft Excel and EViews 12 programs were used to conduct these tests and for the descriptive statistics.

## 3. Results

**Figure 1:**
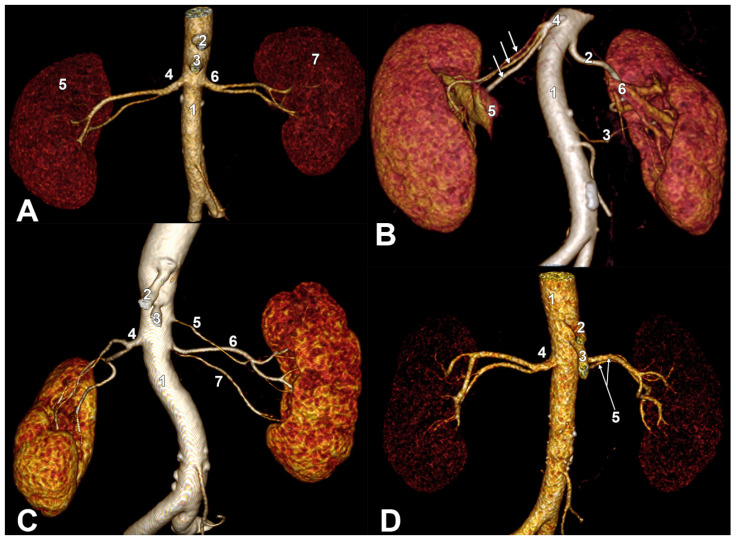
Variants of bilateral combinations of short renal arteries. Three-dimensional volume renderings. Anterior views. (**A**). Bilateral short renal arteries. 1. aorta; 2. celiac trunk; 3. superior mesenteric artery; 4. right (short) renal artery; 5. right kidney; 6. left (short) renal artery; 7. left kidney. (**B**). Short right renal artery (false triple right renal artery). Double left renal artery. 1. aorta; 2. superior left renal artery; 3. inferior left renal artery; 4. right (short) renal artery; 5. right renal vein; 6. left renal vein. The three primary branches of the right renal artery are indicated with arrows. (**C**). Short right renal artery. Right nephroptosis. Triple left renal artery. Right nephroptosis. 1. aorta; 2. celiac trunk; 3. superior mesenteric artery; 4. right (short) renal artery; 5. superior left renal artery; 6. middle left renal artery; 7. inferior left renal artery. (**D**). Short right renal artery (false double right renal artery). 1. aorta; 2. celiac trunk; 3. superior mesenteric artery; 4. right renal artery; 5. double left renal artery.

Short RAs were found in 15/185 cases (8.1%), among which 12/15 were male cases and 3/15 were female cases. In five cases, three males and two females, short RAs were found on the right side (2.7%). In nine cases, eight males and one female, short RAs were found on the left side (4.86%). In one case, bilateral short RAs were found (0.54%) (Figure 1A). The mean length of short RAs in this sublot was 9.46 mm (min. 4.6 mm, max. 14.98 mm, SD: 2.76). The median was 9.42 mm.

**Figure 2:**
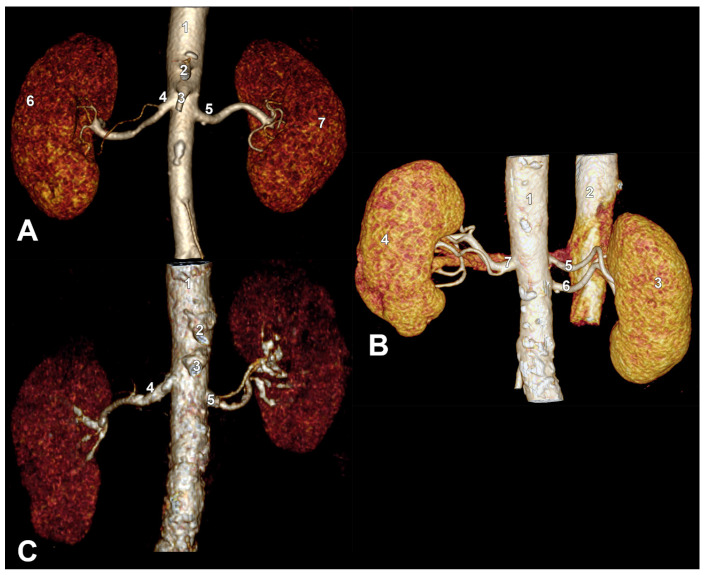
Variants of bilateral combinations of short renal arteries. Three-dimensional volume renderings. Anterior views. (**A**). Short right renal artery with two unequal primary branches. 1. aorta; 2. celiac trunk; 3. superior mesenteric artery; 4. right (short) renal artery; 5. left renal artery; 6. right kidney; 7. left kidney. (**B**). Short left renal artery (false double left renal artery). Double right renal artery. 1. aorta; 2. inferior vena cava; 3. right kidney; 4. left kidney; 5. superior right renal artery; 6. inferior right renal artery; 7. left (short) renal artery. (**C**). Short left renal artery (false double left renal artery). 1. aorta; 2. celiac trunk; 3. superior mesenteric artery; 4. right renal artery; 5. left (short) renal artery.

The bilateral combinations of RA morphologies were heterogeneous. A trifurcated right short RA and double hilar left RAs were found in a female case (Figure 1B). In another female case, we found a bifurcated short right RA and triple hilar left RAs (Figure 1C). In a male case, we found a bifurcated short right RA and double hilar left RAs (Figure 1D). In three cases, two males and one female, there were bifurcated short right RAs and single hilar left RAs (Figure 2A). In a male case, we found double hilar right RAs and a bifurcated short left RA (Figure 2B). In three males, we found single right hilar RAs and bifurcated short left RAs (Figure 2C).

**Figure 3:**
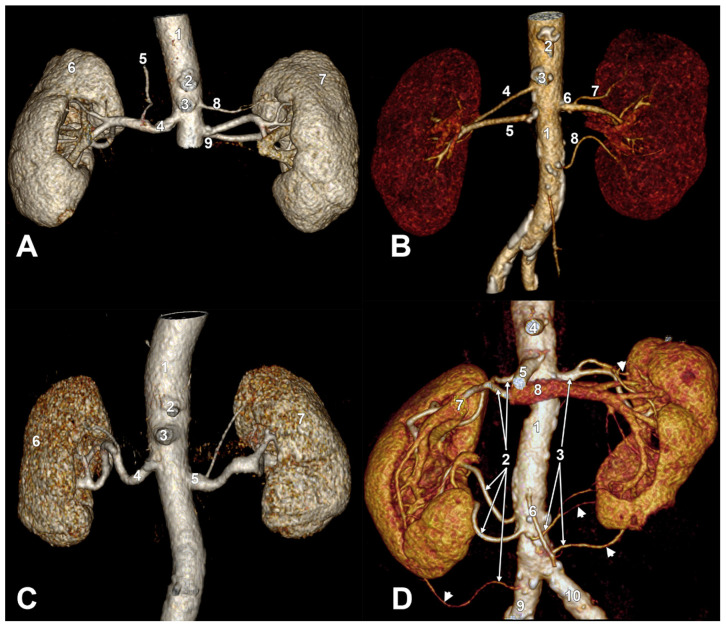
Peculiar variants of bilateral combinations of short renal arteries. Three-dimensional volume renderings. Anterior views. (**A**). Double left renal artery. Short inferior left renal artery. 1. aorta; 2. celiac trunk; 3. superior mesenteric artery; 4. right renal artery; 5. right inferior phrenic artery; 6. right kidney; 7. left kidney; 8. superior left renal artery; 9. inferior (short) left renal artery. (**B**). Bilateral double renal arteries. Short superior left renal artery with a superior polar branch. 1. aorta; 2. celiac trunk; 3. superior mesenteric artery; 4. superior right renal artery; 5. inferior right renal artery; 6. superior (short) left renal artery; 7. superior polar branch; 8. inferior left renal artery. (**C**). Short left renal artery. 1. aorta; 2. celiac trunk; 3. superior mesenteric artery; 4. right renal artery; 5. left (short) renal artery; 6. right kidney; 7. left kidney. (**D**). Malrotated kidneys, dehiscent renal sinuses, bilateral multiple renal arteries. 1. aorta; 2. right renal arteries; 3. left renal arteries; 4. celiac trunk; 5. superior mesenteric artery; 6. inferior mesenteric artery; 7. right renal vein; 8. left renal vein; 9. right common iliac artery; 10. left common iliac artery. The arrowheads indicate polar arteries.

In four other male cases, peculiar morphological patterns of RAs were found (Figure 3). One of these cases had a single right RA, from which left an inferior right phrenic artery. On the left were seen two aortic branches: a right superior polar artery further divided into two superior polar branches entering separately into the upper pole of the left kidney and a short left RA bifurcating into two main left RAs (Figure 3A). The second case had two main RAs on each side. The superior left RA was early-branched into a left superior polar artery and a superior hilar left RA. The second left RA entered the lower angle of the left renal hilum (Figure 3B). The next case had bilateral superior polar arteries. The left one left from a short trunk of the left RA (Figure 3C). The last of these four peculiar cases (Figure 3D) had malrotated kidneys with anteriorly dehiscent renal sinuses. There were five right RAs. The four superior ones were hilar arteries, and the three inferior ones had precaval courses. The fifth was a right inferior polar artery originating from the right common iliac artery. On the opposite side, there were three left RAs. The superior one was early bifurcated into a left superior polar branch and a main hilar left RA from which a second superior polar artery left. The middle left RA was divided into a hilar artery and an inferior polar artery. The inferior left RA was an inferior polar artery.

When the positive case–gender correlations were tested in the general lot, we found that short RAs were not significantly related to any gender (*p* > 0.05). When the positive case–side correlations were tested in the general lot, significant correlations resulted for the right side (*p* < 0.00) and the left side (*p* < 0.00). We found a significant correlation between gender and right short RAs (*p* < 0.05).

## 4. Discussion

Short RAs with a mean length of 9.46 mm were found in 8.1% of cases, either on the right side, left side, or bilaterally (one case). In most cases, short RAs were bifurcated. Just one right RA was trifurcated. Renal arterial anatomy differed between sides [7]. Therefore, the bilateral combinations of RA morphologies were heterogeneous in cases with short RAs. In 15/185 cases, multiple hilar RAs and different morphologies of renal polar arteries were also found. Complex bilateral morphologies of RAs were also encountered. Short RAs may appear on any side.

Different studies have evaluated the prevalence of early-branched RAs [13,16,17,18]. Some authors defined the early branching of an RA as segmental branches originating from it more proximal than the level of the renal hilum [18]. In this regard, early branching was regarded as prehilar branching. Later, Kumaresan et al. (2022) distinguished between these two patterns [16]. Other authors used the terms peri-hilar or extra-parenchymal branching of RAs but overlooked the early branching of RAs when examining renal angiograms [19]. Gümüş et al. (2012) classified the variations in RAs into two groups: extrarenal RAs and early divided RAs [20]. Extrarenal RAs were either polar or accessory hilar arteries [20]. These authors did not refer to prehilar branching of RAs because they regarded early-divided RAs as having segmental divisions more proximal than the renal hilus level [20]. This, although they documented other studies and defined an early-branched RA as a variant in which “any branch diverges within 1.5–2.0 cm from the lateral wall of the aorta in the left kidney or in the retrocaval segment in the right kidney” [20].

Kumaresan et al. (2022) distinguished 8 cardinal and 10 minor perihilar branching patterns [16]. The duplicated fork morphology (type I) of perihilar branching was the most frequent (70.2%) among 198 kidneys [16]. They found early-divided RAs in 20.2%, but we could not see how the early branching of RAs was defined in that study [16]. However, these authors distinguished between early branching and prehilar branching of RAs.

Munnusamy et al. (2016) found 13/100 cases with early divided RAs, five on the right, seven on the left, and one bilateral [13]. In this last study, if the main RA was divided into segmental branches within 1 cm from its origin, it was considered an earlier division [13]. The authors did not perform statistical tests. Kumaresan et al. (2022) found early divided RAs in 20/99 cases (20.2%) [16]. The statistical analysis in that study revealed that there was no association between gender and laterality in the prevalence of early division of renal arteries (*p* = 0.08) [16]. We found a significant correlation between gender and right-sided short RAs (*p* < 0.05). Çinar and Türkvatan (2016) classified any RA <1.5 cm as “prehilar (early) branching” [17]. They found this pattern in 33/504 patients (6.5%), of which 13 were on the right side, 19 were on the left side, and one was bilateral [17]. In 11/504 cases (2.2%), Çinar and Türkvatan (2016) found both accessory RAs and early-branched RAs [17]. They did not find any association between the existence of RA variations and gender (*p* = 0.630) [17].

Recto et al. (2019) reviewed 64 articles (20.782 kidneys) for variations in RAs [14]. As the authors found, early branching patterns were described in only one-third of the published data, being present in 11.4% of the total kidneys from these data (4.23% of right RAs, 4.52% of left RAs, and 2.66% had no right/left information) [14]. Rao et al. (2006) reported a case with bilateral early-branched RAs [21].

Covantsev et al. (2018) studied 28 pairs of kidneys by dissection and 93 aortographies [22]. They indicated that an RA can branch early, but no distinction was made between early and prehilar branching [22]. These authors found that early bifurcation and trifurcation of the arteries were more often found on the right [22]. Tarzamni et al. (2008) found early-branched RAs in 35.98% of cases [23]. Although these authors did not distinguish between early and prehilar branching or measure lengths of RAs, they found no significant differences between early-branched right and left RAs (*p* = 0.264) [23]. Stojadinovic et al. (2022) considered RAs <1.5 cm as early-divided (short) and found prevalences of 5.5% on the right side and 7.3% on the left side [24]. We found short right RAs in 2.7%, short left RAs in 4.86%, and bilateral short RAs in 0.54%. Natsis et al. (2014) found short RAs (<1.5 cm) in 30.4% (7/23) of kidneys [3]. Aremu et al. (2021) found <1 cm short early-branched RAs in 18% of 100 consecutive living kidney donors [25].

We found bifurcated and trifurcated short RAs in different bilateral combinations with opposite multiple RAs. These morphologies result from fetal development: either one of several mesonephric arteries persists to form a single RA, or, if the fusion of the urogenital rete arteriosum is not fully completed, short RAs or multiple RAs may result [24].

The anatomical variations in renal vessels are essential in surgery, nephrology, and radiology [26]. In any surgical and interventional procedures, including renal transplants, aneurysmorraphy, and other vascular reconstructions, unawareness of the presence of multiple RAs may result in a fatal outcome [26].

In this study, double, triple, and pentuple RAs were found. Their prevalence was not determined in the general lot. However, pentuple RAs are a rare anatomic variation. We found such a case here. The kidneys were malrotated (non-rotated), with dehiscent renal sinuses broadly opened anteriorly. We could not find specific information when searching for the term “dehiscent renal sinus”. Such a case with bilateral malrotation of kidneys, dehiscent renal sinuses, and multiple renal vessels was found during dissection [27]. The renal sinus dehiscence was indicated as “opened hilum” [27]. We agree with the authors that anomalies like renal malrotation and dehiscent renal sinus are infrequently reported [27]. Buffoli et al. (2015) found a malrotated right kidney at dissection with three precaval right RAs [28]. We found a malrotated right kidney with five RAs, of which the three inferior ones had precaval courses. Knowledge of possible renal rotational variations is vital, especially in interpreting intravenous pyelograms [29]. Abrupt narrowing of the renal pelvis and angular termination of the renal calyces into it may lead to hydronephrosis, especially when neighboring organs press on it [29]. Narrowing of the renal pelvis may lead to entrapment of renal calculi [29]. The renal rotation precedes the definitive vascularization of the kidneys and occurs during the ascent of the kidney [30]. The developing metanephros shifts laterally, and the renal hilum rotates from anterior to medial [30]. In non-rotation, the renal hilum is oriented anteriorly, and in incomplete rotation, it is antero-medial [30].

Triantafyllou et al. (2024) worked on a systematic review and meta-analysis of accessory RAs [4]. They documented the pooled prevalence of multiple RAs (Table 1). They found just six cases with five RAs in the literature [4]. Subgroup analyses revealed no significant differences in accessory RAs in geographic distribution, type of study, sample size, side, or sex [4].

In one of the cases with short left RAs, the right inferior phrenic artery (RIPA) left from the right RA. This variant of origin of the RIPA was found in 10.4% of cases by Aslaner et al. (2017) [31]. Hiwatashi and Yoshida (2003) found and confirmed it in 2/26 patients [32], while Basile et al. (2008) found it in 5.5% [33]. The RIPA is the most common extrahepatic collateral pathway supplying hepatocellular carcinomas [33].

Here, we found different morphological possibilities of renal polar arteries: superior or inferior, with aortic, renal, or iliac origins, and single or dichotomized. Such possibilities are known and were specifically studied previously [9]. During renal transplantations, polar vessels can increase the failure rate because of urinary leakage and thrombosis of the renal vessels [9].

During development, the arterial supply of the kidney shifts from the common iliac artery to the abdominal aorta [2]. It is, therefore, common to find an iliac origin of an additional inferior RA. In the case with five right RAs, the inferior one was an inferior polar artery with a precaval course originating from the common iliac artery. Tardo et al. (2017) found a right inferior polar RA originating from the common iliac artery in 300 subjects (0.34%) [34]. However, they did not supply specific evidence or mention whether the inferior polar right RA had a precaval or retrocaval course. Identifying precaval right RAs is crucial when planning minimally invasive renal approaches [35].

A renal segmental artery is considered a second or third-order RA branch [36]. Therefore, identifying such extrarenal branches of RAs and their specific distribution to renal segments will remain elusive if they are not cautiously documented.

A limitation of this anatomical study is that at the time when the archived angioCT scans were used, the functional parameters of these cases were not available, nor did we aim to document them. Also, as it is an anatomical study, the cases we used were not documented clinically.

## 5. Conclusions

During renal transplant surgery, distinguishing between true and false multiple RAs is essential. While true multiple RAs may cause surgical discomfort, short RAs may be used as single RAs, but they should be carefully documented before donor nephrectomy.

## Figures and Tables

**Table 1:** The pooled prevalence of multiple renal arteries, from [4].

Number of Renal Arteries	Pooled Prevalence
2	18.67% (95% CI: 16.72–20.69%), bilateral in 5.15% (95% CI: 3.82–6.64%). The pooled prevalence of left-sided double RAs was estimated at 18.94% and right-sided double RAs at 18.60% (*p* = 0.88).
3	1.80% (95%CI: 1.34–2.32), bilateral in 0.30% (95% CI: 0.06–0.66%). Three RAs on the left had a pooled prevalence of 2.12% and, on the right, a pooled prevalence of 1.91% (*p* = 0.71).
4	0.01% (95% CI: 0.00–0.04%), and fewer than 0.01% were bilateral (95% CI: 0.00–0.11).
5	<0.01% (six cases reported previously)

## Data Availability

The original contributions presented in this study are included in the article. Further inquiries can be directed to the corresponding author.
